# Treatment of Vesicoureteral Reflux after Puberty

**DOI:** 10.1155/2008/590185

**Published:** 2009-02-26

**Authors:** J. Christopher Austin

**Affiliations:** Depatment of Urology, University of Iowa, 200 Hawkins Drive, Iowa City, IA 52242, USA

## Abstract

Vesicoureteral reflux is uncommonly diagnosed and treated after puberty. The natural history of uncorrected VUR after puberty is not documented. Postpubertal patients with recurrent pyelonephritis and VUR should be considered for treatment. Ureteral reimplantation, endoscopic injections, and laparoscopic or robotic ureteral reimplantation may be utilized. Endoscopic injection is an appealing option for these patients. The role of laparoscopic or robotic ureteral reimplantation in these patients is evolving.

## 1. INTRODUCTION

Vesicoureteral reflux (VUR) is a common finding in children with urinary tract infections (UTIs). The incidence of VUR associated with UTIs drops significantly in older children, particularly after the age of 5 [1]. It has been a long-held dogma of pediatric urology that unresolved VUR should be treated before a child progresses through puberty. There is concern that females with uncorrected VUR will have problems with pyelonephritis when they become sexually active and during pregnancy. Pyelonephritis during pregnancy increases the risks of the pregnancy and possibly to the unborn child as well. If these children have their reflux corrected before they go through puberty, these problems could be prevented. While in theory this justifies the surgical correction of all children who fail to resolve their reflux during a period of observation, there is little evidence to support treating all children and no long-term studies documenting the natural history of uncorrected VUR after puberty. This manuscript will review the association of VUR and UTIs in patients treated after puberty and examine the role, nuances, and outcomes of treating VUR after puberty.

## 2. CLINICAL IMPORTANCE OF VUR AFTER PUBERTY

There has been a long-standing observation of the association of febrile UTIs and the development of renal scarring [2]. The riskiest time for the development of renal scarring due to febrile UTI associated with primary VUR is infancy and the risk of developing new scars drops significantly after age of 5 [3]. It is still common to follow and treat patients beyond this age. While severe scarring may predispose to chronic kidney disease in a small percentage (estimated at 2%), the majority of children with VUR are not at significant risk for renal failure [4]. There is a higher incidence of hypertension in patients with renal scarring, but severe complications due to VUR are unusual. The only reliable benefit to patients who's VUR is surgically corrected is a decreased risk of pyelonephritis, but the incidence of UTIs is similar to those who reflux has persisted. These findings have led some to consider stopping antibiotic prophylaxis in selected children and ceasing surveillance for resolution [5, 6]. Voiding cystourethrograms (VCUG) can be perceived as traumatic by children and simplifying their follow-up and avoiding these tests may be a major factor in discontinuing follow-up for some parents. As a result of these practices more and more physicians are going to allow children with persistent VUR to continue through puberty with uncorrected VUR. As a group, these patients will likely have lower grades of VUR, infrequent UTIs, and normal renal function. There are currently no longitudinal studies documenting just what risks these patients will face and whether their VUR will be a significant problem during adolescences and adulthood. It is likely that some of these patients will have future problems with UTIs and be considered for treatment of their VUR after puberty.

There has also been a change in VUR management by some pediatric urologists that is the polar opposite of stopping prophylaxis and observing patients. Instead of following patients for resolution of their VUR, primary therapy with endoscopic injections after the diagnosis of VUR is being offered [7]. Touted as a minimally invasive alternative, the “15-minute cure” is being used as upfront therapy in patients after diagnosis to avoid the need to prolonged antibiotic prophylaxis and prolonged follow-up while waiting for the VUR to resolve. While success rates greater than 90% are reported by experienced surgeons, the follow-up in these series is generally short with VCUGs being performed usually within 3 months of the procedure [8]. There is a known rate of relapse for this method which is highest for collagen, and was reported to be greater than 50% at 5 years [9]. The relapse rate is lower for other agents [10, 11]. If there is a 10–15% relapse over 5–10 years (a reasonable estimate given the few long-term studies available) it is likely that there will be increasing numbers of late failures of endoscopic therapy. Of these patients, some will present after puberty with pyelonephritis and recurrent VUR.

## 3. VUR AND UTIs AFTER PUBERTY

The primary motivation for treatment of VUR before puberty has been over concerns of increased risk of UTIs when female patients become sexually active or pregnant. To my knowledge, however, the natural history of women with persistent VUR during pregnancy has not been documented. Pyelonephritis is a known risk to pregnant women and strongly associated with bacteruria. Large series have reported rates of 2% of pregnant women [12]. In pregnant women with a history of VUR or surgically corrected VUR, higher rates of UTIs and pyelonephritis have been reported. Mansfield et al. reported complications during pregnancy for patients who had undergone surgical correction of VUR and a group with a history of VUR that did not undergo surgery [13]. The rate of cystitis and pyelonephritis was 22% and 18%, respectively, during pregnancy for the patients with surgically corrected VUR. These rates were higher than the patients without treatment of their VUR with cystitis in 15% and pyelonephritis in 1.5%. They also reported a high rate of UTIs with the onset of sexual activity in 75% of patients with surgically treated VUR and 62% of those with a history of VUR that was not treated. There is a significantly increased risk of UTIs in women with a history of VUR. There could be multiple explanations for the differences in the two groups including that the surgical procedure is the cause of the increased UTIs. Due to the retrospective nature however, the authors point out that this observation of higher rates of UTIs in the surgically treated patients does not prove that the problem is due to the surgery. Other factors may be responsible for these differences, such as selection bias. These patients likely had an increased susceptibility to UTIs, which is likely what pushed them to surgical correction in the first place. The authors recommend that pregnant women with a history of ureteral reimplantation surgery undergo frequent screening for bacteruria during pregnancy and that prophylactic antibiotics be considered in these patients. Other series of long-term follow-up of patients with VUR treated with ureteral reimplantation surgery have similar findings. Beetz et al. reported a 25-year follow up in 158 patients [14]. Female patients reported subsequent UTIs in 74% after surgery versus 10% of males. 17% developed UTIs during pregnancy, however, they did not report whether these patients had pyelonephritis or cystitis. Of the UTIs, most (66%) were afebrile versus preoperatively where the UTIs were febrile. It remains unknown as to whether patients experiencing continued problems with UTIs after surgery would have fewer problems if they had not undergone surgery.

## 4. WHICH PATIENTS SHOULD BE EVALUATED AND TREATED?

The incidence of VUR after puberty is significantly lower than in young infants with febrile UTIs [1]. It has been well established that VUR is a risk factor for pyelonephritis and that it frequently is present as one of several risk factors for UTIs. Voiding dysfunction must always be considered in this patient population and treated appropriately. With proper treatment and modification of risk factors such as infrequent voiding, dysfunctional voiding, constipation, and incomplete emptying the VUR may be eliminated without surgical treatment [15].

Most postpubertal patients with VUR will present with UTIs. Since the majority of patients will not have VUR and assuming that VUR is an important risk factor for pyelonephritis, what are the factors that should prompt an evaluation for VUR in a postpubertal patient? First and foremost should be a history of recurrent febrile urinary tract infections, as these patients if found to have reflux, have a good chance of having their symptoms alleviated if VUR is found and treated. Suspicion should be raised if there is a history of prior VUR, febrile infections as a young child, or a family history of VUR. Due to the high association of renal scarring and VUR, patients who have a history of recurrent febrile UTIs and evidence of renal scarring on imaging studies should be evaluated for VUR. To evaluate for VUR a standard VUCG may be performed, or if there are symptoms worrisome for voiding dysfunction a videourodynamics study. In patients with evidence of renal scarring or a small kidney on ultrasound, a DMSA renal scan should be obtained to evaluate for renal scarring and the differential function. Typically, the grade of VUR is an important factor for predicting resolution and risk of renal scarring; however, in postpubertal patients it has not been studied as extensively. It is expected that the rates of spontaneous resolution will be lower after puberty.

## 5. SURGICAL TREATMENT OF VUR AFTER PUBERTY

One of the more humbling experiences for a pediatric urologist is their first ureteral reimplantation done on a female after puberty. These difficulties start as females approach puberty. During puberty, the pelvis widens and deepens in the female. The trigone assumes a deeper retropubic location which makes access to the ureteral orifices and the mobilization of the ureters more difficult. Additionally, the plexus of vein running across the surface of the bladder enlarge and are more prone to troublesome bleeding during ureteral dissection. Though not well documented, most pediatric urologists would agree that, if a child truly needed ureteral reimplantation for correction of their VUR, then the operation is best performed if they are operated on during childhood rather than after puberty. Experience would tell us that they recover quicker and that technically the surgery should be more successful, however, there are not series documenting poorer results after ureteral reimplantation for patients treated after puberty. Options for treatment of VUR in patients after puberty include intra- or extra-vesical ureteral reimplantation, endoscopic injection, and laparoscopic or robotic reimplantation. In patients with unilateral VUR to a poorly functioning kidney nephrectomy may be an alternative choice to ureteral reimplantation. This can be performed laparoscopically with rapid recovery and short hospitalization.

## 6. URETERAL REIMPLANTATION

As mentioned previously, the postpubertal changes in women make the surgical access to the trigone more challenging and limit exposure; however, ureteral reimplantation can be performed in women successfully. In males, the changes are less dramatic. Published results of ureteral reimplantation in postpubertal patients are sparse due to the limited number of patients in whom this surgery is indicated. From a technical standpoint, it is prudent to position the patient over the break in the OR table. This allows the table to be flexed if necessary to open the pelvis and improve retropubic exposure. This is similar to the positioning of a male undergoing radical retropubic prostatectomy. The size and body mass index will play a role with obese patients creating more difficulties with exposure. Proper surgical planning and the use of larger and more flexible fixed retractors such as a Bookwalter rather than the Denis-Browne retractor will facilitate the procedure. Both ureteral advancements and ureteroneocystotomy procedures (e.g., Cohen, Glenn-Anderson, and Politano-Leadbetter) can be performed but limited data is available on their use in the treatment of VUR postpuberty [16–20]. There have been reports of the use of trigonoplasty (Gil-Vernet) procedures, which in postpubertal patients offer the advantage of less dissection and mobilization [21, 22]. Success rates of up to 97% have been reported but follow-up is limited to only 11 months.

## 7. ENDOSCOPIC INJECTION

Although controversy remains about the role of endoscopic injection for VUR as an alternative to ureteral reimplantation in young children, there are few “reimplanters” who would completely dismiss this option in patients after puberty. This technique has been practiced for over 20 years and a variety of bulking agents have been injected including polytetrafluoroethylene paste, gluteraldehyde cross-linked bovine collagen, polydimethysiloxane, and detranomer/hyaluronic acid copolymer (D/HA) [23]. In the United States the D/HA copolymer is the only agent that has been FDA approved for use in children. A recent series by Okeke et al. reported 9 women (mean age of 26) with symptomatic VUR associated with acute pyelonephritis who were treated endoscopically with D/HA [24]. All were treated successfully with no reflux by VCUGs at 3 months postoperatively, although 1 did require a second injection for persistent VUR. Two patients had transient flank pain immediately postoperatively which resolved after a few days. At a mean follow-up of 14 months, none of the patients had further infections or symptoms. In children, the treatment of VUR with D/HA injections has been associated with a lower incidence of UTIs postoperatively [25]. It is unknown whether or not postpubertal patients are at the same risk for late failures of endoscopic injection as was described in some of the original series of children.

## 8. LAPAROSCOPIC AND ROBOTIC URETERAL REIMPLANTATION

Minimally invasive ureteral reimplantation for VUR has been performed for over a decade; however its widespread uses have been slow to spread. Proponents of laparoscopic surgery would cite the limited laparoscopic experience of most pediatric urologists as a primary factor for this slow adaptation. However, many would argue against relearning to do a procedure which has rapid recovery, high success rate, low complication rate, and is done through an inconspicuous incision. A low, transverse abdominal incision leaves a scar that often blends into the natural creases of the abdominal skin leaving a barely visible mark. If placed low enough it is covered by undergarments and is not disfiguring. Given the questionable benefits and technical difficulties the laparoscopic technique has been slow to gain acceptance. One population where this approach actually may be a real asset is in treating VUR after puberty.

Both intra- and extra-vesical laparoscopic techniques for reimplantation have been described in children [26–28]. In the postpubertal female, the advantage for laparoscopy is that the scope and instruments can reach deep into the pelvis with good visualization. Shu et al. reported a series of laparoscopic extravesical reimplantations performed for VUR in postpubertal females at a mean age of 18 [29]. One patient underwent bilateral and the other 5 unilateral reimplantation. 1 patient had transient ureteral obstruction requiring a temporary stent placement. All had resolution of their VUR postoperatively.

Robotic assisted laparoscopic surgery has become a widely practiced urologic technique for performing radical prostatectomy [30]. The surgeons utilizing the robot for prostatectomies tout the increased magnification, 3D visualization, and precision movements the wristed instruments as advantages over the open surgical procedure. The neurovascular bundles are well visualized, as is the apical dissection of the prostate and urethra. Performing deep pelvic surgery such as ureteral reimplantation in postpubertal patients is a situation where the robotic approach may have an advantage over traditional open surgical reimplantation. There are limited reports of robotic ureteral reimplantations being performed in children [31]. The robot allows a surgeon with good open surgical skills to perform complex laparoscopic procedures. Extravesical ureteral reimplantation has been associated with postoperative urinary retention when performed bilaterally [32]. This is felt to be due to injury to the pelvic plexus during the dissection of the ureter extravesically [33]. Casale et al. reported a series of 41 children with a mean age of 33 months treated with bilateral robotic “nerve-sparing” extravesical reimplantation [34]. All children voided well after catheter removal on postoperative day number 1. No patient had retention as documented by ultrasonic bladder scanning. Reflux was cured in 97% and no ureters were obstructed. If these impressive results can be achieved by other surgeons and applied to postpubertal patients, this could become the preferred approach to ureteral reimplantation surgery after puberty.

## 9. CONCLUSIONS

The natural history of untreated VUR in postpubertal patients is unknown. Treatment of VUR should be considered in those patients with VUR and recurrent febrile UTIs. The optimal method to treat VUR is not clear. Endoscopic injection is a minimally invasive approach which has a good success rate for treating VUR and offers some benefits over ureteral reimplantation. The role of laparoscopic and robotic ureteral reimplantation is evolving and may offer some advantages over open ureteral reimplantation in postpubertal patients.
